# Risk factors for refractory *Mycoplasma pneumoniae* in Chinese children: a meta-analysis

**DOI:** 10.3389/fped.2025.1512689

**Published:** 2025-06-27

**Authors:** Chun Chen, Shan Chen, Chuanzhong Yang, Xiaolan Zhang, Luohui Liu, Yuejuan Wang, Min Cao

**Affiliations:** ^1^ Department of Neonatology, Shenzhen Maternity & Child Healthcare Hospital, Shenzhen, China; ^2^ Department of Emergency, Shenzhen Maternity & Child Healthcare Hospital, Shenzhen, China

**Keywords:** refractory *mycoplasma pneumoniae*, child, children, riskfactors, meta-analysis

## Abstract

**Background:**

With the increase of the incidence rate of *Mycoplasma pneumoniae* in children and the widespread use of azithromycin, the number of cases of refractory *M. pneumoniae* increased accordingly. *M. pneumoniae* infection was generally considered a self-limiting disease. However, under certain special circumstances, it was highly likely to develop into a refractory disease. This study conducted a meta-analysis of early risk factors for refractory *Mycoplasma pneumoniae* pneumonia (RMPP), which was helpful for the early clinical diagnosis of RMPP and the reduction of sequelae.

**Methods:**

This systematic search was conducted in Web of Science, Embase, PubMed, Cochrane Library, CNKI, Wangfang, Sinomed and Cqvip, and the date was set until August 20, 2024. After two researchers independently screened the literature, extracted data, and assessed the risk of bias in the included studies, a meta-analysis was conducted using STATA 17.0 and RevMan 5.4 software.

**Results:**

Twenty-eight studies including 6374 patients were included in this analysis, and the results showed that the age [*MD (95% CI)*: 0.62 (0.21, 1.03), *P* = 0.003], LDH [*MD (95% CI*): 161.57 (128.22, 194.91), *P* < 0.001], neutrophils (%) [*MD (95% CI*): 9.27 (3.45, 15.09), *P* = 0.002], IL-6 [*MD (95% CI*): 23.07 (20.90, 25.24), *P* = 0.04], ESR [*MD (95% CI*): 10.93 (7.75, 14.11), *P* < 0.001], AST [*MD (95% CI*): 16.11 (8.21, 24.01), *P* < 0.001], ALT [*MD (95% CI*): 23.69 (9.60, 37.77), *P* = 0.001], CRP [*MD (95% CI*): 23.72 (18.41, 29.03), *P* < 0.001], and WBC [*MD (95% CI*): 1.07 (0.28, 1.86), *P* = 0.008] were higher in the RMPP group than in the NRMPP group. Combined pleural effusion (*OR* = 7.59, *95% CI*: 4.19–13.75, *P* < 0.001) and lung consolidation (*OR* = 10.61, *95% CI*: 4.13–27.26, *P* < 0.001) were identified as risk factors for RMPP. However, no significant association was found between gender and the incidence of RMPP (*OR* = 0.91, *95% CI*: 0.80–1.02, *P* = 0.10). The analysis of publication bias indicated that 3 of the 11 factors analyzed [LDH, neutrophils (%), and lung consolidation] showed significant publication bias (*P* < 0.05).

**Conclusion:**

Our study further confirmed that elevated inflammatory markers such as CRP, LDH, neutrophils (%), IL-6, ESR, lung consolidation, combined pleural effusion were risk factors for RMPP. For the first time, WBC, ALT, and AST were identified as risk factors for the occurrence of RMPP in children. Additionally, demographic information such as age and gender was also examined in relation to RMPP in children.

## 1 Introduction

*Mycoplasma pneumoniae*, an obligate intracellular pathogen without a cell wall, had various virulence factors enabling it to overcome host defenses. Its infection triggered immune responses like leukocyte pro-inflammatory effects and epithelial cell metabolic changes, causing severe respiratory symptoms in humans, particularly children (1, 2). *M. pneumoniae* infections could also lead to extrapulmonary manifestations in multiple systems (3, 4). RMPP occurred when patients still had fever, persistent symptoms, worsening lung imaging and even extrapulmonary complications after 7 days of macrolide antibiotic treatment. Severe cases might endanger pediatric patients' lives and lead to complications like obliterative bronchitis, bronchiectasis and interstitial lung disease, which could reduce exercise tolerance and quality of life (5). Due to its high antibiotic resistance rate, while vaccination was being studied (6), early RMPP recognition via clinical indices was crucial for pediatric clinicians to shorten the disease course, alleviate family economic burdens and reduce children's suffering. The high RMPP incidence in Asia had made it a clinical research hotspot (7). Studies had indicated that LDH, CRP, neutrophils (%), D-dimer, and ESR were RMPP risk factors (8–10), but some research had found contradictory results (11). The elusive pathogenesis of pediatric RMPP underscored the need to study risk factors for early high-risk identification and management, given its rising incidence, multifarious complications, and suboptimal outcomes. This meta-analysis investigated RMPP risk factors to provide evidence-based clinical guidance for early intervention.

## 2 Methods

### 2.1 Databases

English databases included PubMed, Embase, Cochrane Library, and Web of Science. Chinese databases include China National Knowledge Infrastructure (CNKI), Wanfang, Cqvip, and SinoMed. The search time was set from the establishment of each database to August 20, 2024. We selected literature that had publicly publishes research on the risk factors of pediatric refractory *M. pneumoniae* pneumonia.

### 2.2 Search strategies

The retrieval strategy adopted a combination of subject headings and free text keywords, adjusted according to the characteristics of each database. The search method was “*M. pneumoniae* pneumonia” OR “MPP” OR “refractory *M. pneumoniae* pneumonia” OR “RMPP” AND “children” OR “child” AND “factor” OR “risk”. Taking the PubMed search strategy as an example, it was presented in Supplementary Table S1.

### 2.3 Inclusion and exclusion

Inclusion criteria:

(1) The types of study included cohort studies or case-control studies.
(2) The case group consisted of children with RMPP, while the control group consisted of children with typical *M. pneumoniae* pneumonia.
(3) Articles in Chinese (from core journals) and articles in English.

Exclusion criteria:

(1) studies with duplicate publications;
(2) conference abstracts, reviews, case reports, meta-analyses or editorials;
(3) studies with incomplete data records;
(4) animal experimental research.

### 2.4 Data extraction

Two researchers separately conducted literature screening, data extraction, and peer—checking. If conflicting data emerged, a third researcher made a judgment. The literature screening process was as follows: ① Screen titles to exclude obviously irrelevant literature; ② Read abstracts and full texts to determine inclusion. They used EXCEL to extract key information, including the first author, publication year, study region, research year, literature type, total number and age of case/control groups, and outcome indicators.

### 2.5 Quality assessment

The Newcastle-Ottawa Scale (NOS) was employed to evaluate the quality of the eligible observational studies. The NOS is a composite assessment strategy that provides one score for each study based on questions related to 3 key domains: selection bias, comparability for assessment of confounding, and outcome/exposure definition. Standard questionnaires are available for cohort and case–control studies. The total score of NOS was 9 points, and the research quality was divided into low quality (0–3 scores), medium quality (4–6 scores), and high quality (7–9 scores). The details of methodological quality assessment of included studies were showed in Supplementary Table S2.

### 2.6 Statistical analysis

Forest plots were used to display effect sizes and confidence intervals for each outcome in the meta-analysis, as well as the overall summary effect. Heterogeneity testing assessed various indicators. When the heterogeneity statistic I^2^ was less than 50%, the fixed effects model was adopted; Otherwise, random effects model was used. Binary variables used odds ratio (OR) as the effect analysis statistic, continuous variables used mean difference (MD) as the effect analysis statistic, and provide a 95% confidence interval (CI) was provided for each effect size. Sensitivity analysis was conducted on the model, and publication bias was detected through Egger's test. The difference was considered statistically significant when *P* < 0.05. When the number of included studies based on outcome indicators was ≥10, funnel plots were used to analyze publication bias. Meta-analysis was conducted using STATA 17.0 software and Review Manager version 5.3.0.

## 3 Results

### 3.1 Literature screening process

**Figure 1:**
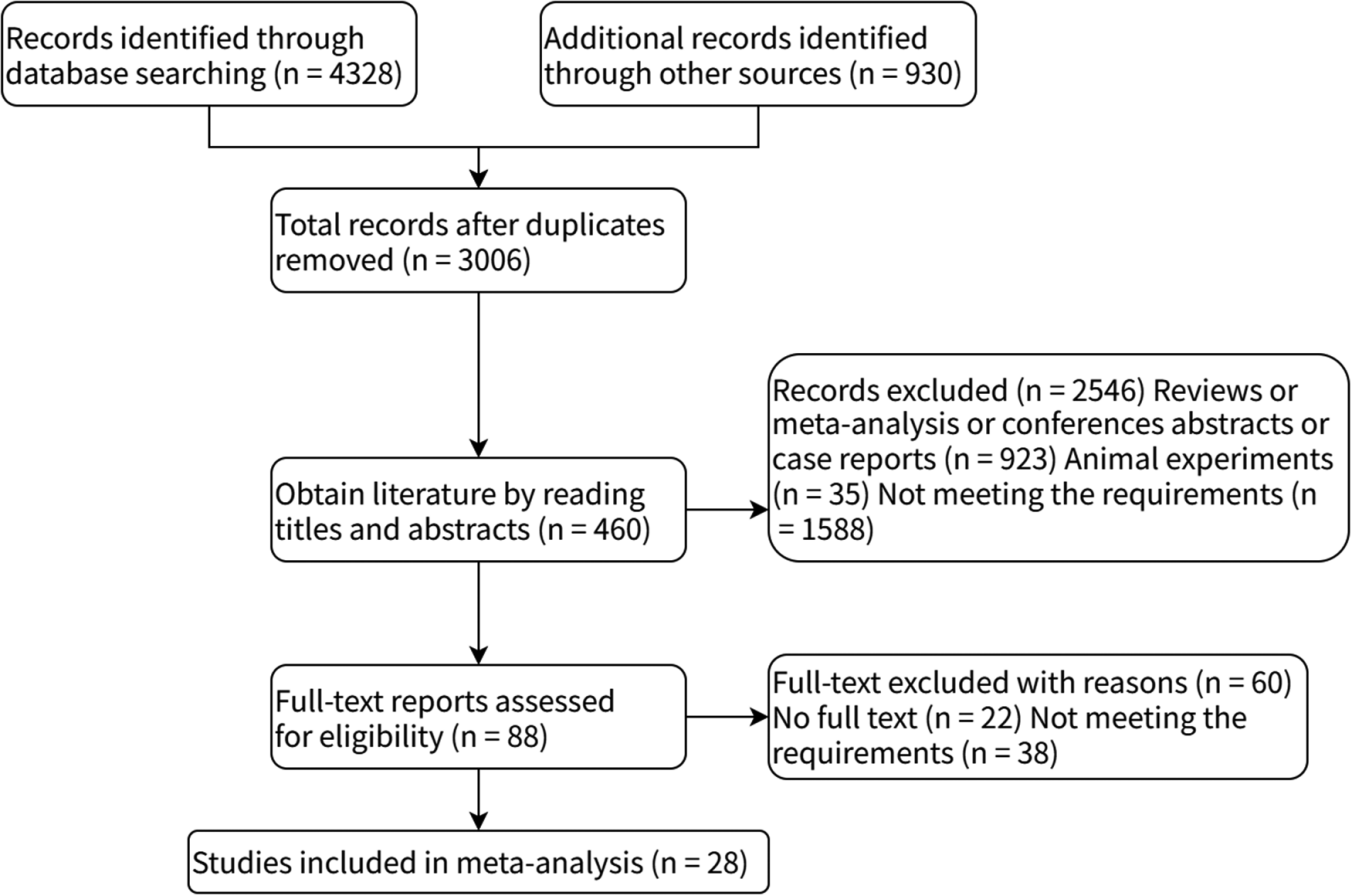
Retrieval flow chart.

A total of 5,258 articles were retrieved. After removing duplicate literature, 3,006 articles remained, and 28 studies were selected based on inclusion and exclusion criteria. Figure 1 illustrates the detailed literature search process.

### 3.2 Basic feature

The included literature types were case-control studies published between 2012 and 2024, with a total sample size of 6,374 cases. There were a total of 23 high-quality studies (9, 12–32) and 5 medium-quality studies (33–37). The results of the literature quality evaluation were shown in Table 1, and the characteristics of the studies were shown in Table 2.

**Table 1:** Baseline characteristics.

Author	Publication year	Study year	Total		Age, years		Quality assessment	Grade of evidence
			E	C	E	C		
Xu et al. (25)	2024	2019–2020	55	391	5.0	5.2	8	High
Chen et al. (28)	2024	2021–2022	156	312	6.2 ± 2.9,	5.6 ± 3.0	9	High
Li et al. (32)	2024	2018–2022	56	70	5.1 ± 3.4,	4.9 ± 3.1	7	High
Li et al. (31)	2023	2020–2021	120	109	7.3 ± 2.1,	7.4 ± 2.3	7	High
Wei et al. (24)	2023	2019–2021	70	70	4.9 ± 1.7,	5.5 ± 2.0	8	High
Gao et al. (24)	2023	2019–2021	21	42	5.5	4.2	7	High
Zhang et al. (30)	2023	2017–2018	35	30	4.8 ± 2.6	4.6 ± 2.9	8	High
Li et al. (17)	2023	2018–2021	131	386	6.0	4.8	7	High
Fu et al. (21)	2022	2019–2021	82	156	5.9 ± 2.4	5.5 ± 2.3	8	High
Su et al. (37)	2022	2021	41	101	2.6	2.0	6	Medium
Shen et al. (29)	2022	2018–2021	156	143	6.8 ± 2.3	7.2 ± 2.2	7	High
Huang et al. (9)	2022	2019–2021	31	17	4.8 ± 3.5	4.8 ± 3.2	8	High
Li et al. (15)	2021	2016–2020	125	125	—	—	7	High
Wen et al. (19)	2021	2019–2020	88	216	6.5 ± 2.5	6.1 ± 2.4	8	High
Huang et al. (20)	2021	2019	22	50	1.7	2.2	7	High
Huang et al. (9)	2021	2015	124	306	5.7 ± 2.7	4.3 ± 2.5	7	High
Zheng et al. (12)	2020	2013–2019	73	146	6.5 ± 2.5	6.4 ± 2.8	7	High
Guo et al. (14)	2020	2014–2018	94	60	6.4 ± 1.3	6.3 ± 2.1	7	High
Sun et al. (22)	2020	2016–2018	36	53	6.9 ± 2.0	7.2 ± 1.8	8	High
Li et al. (33)	2019	2018	58	166	6.3 ± 2.7	3.4 ± 1.3	6	Medium
Guo et al. (35)	2019	2017–2018	220	80	6.1 ± 1.6	5.6 ± 1.4	6	Medium
Zhai et al. (13)	2017	2012–2016	142	486	6.8 ± 2.5	4.6 ± 1.9	8	High
Li et al. (16)	2017	2013–2016	92	161	6.5 ± 2.8	5.5 ± 2.5	7	High
Yao et al. (26)	2016	2013–2014	29	68	5.3 ± 3.0	4.4 ± 2.9	7	High
Shao et al. (36)	2015	2013–2014	35	158	—	—	6	Medium
Wang et al. (23)	2015	2014–2015	32	110	6.9 ± 2.8	5.7 ± 2.8	7	High
Lu et al. (18)	2014	2012–2013	353	300	6.9 ± 2.8	5.7 ± 2.8	8	High
Liu et al. (34)	2012	2006–2012	72	141	—	—	6	Medium

**Table 2:** Publication bias.

Factors	*t* value	*P* value
LDH	−6.85	<0.001
WBC	0.54	0.592
IL-6	−1.88	0.061
AST	−0.84	0.400
ALT	1.92	0.055
Neutrophils (%)	−2.27	0.023
CRP	−0.90	0.371
ESR	0.07	0.948
Fever duration	0.11	0.915
Lung Consolidation	−2.81	0.005
Combined pleural effusion	−1.07	0.285

### 3.3 Meta-analysis results

#### 3.3.1 The age

**Figure 2:**
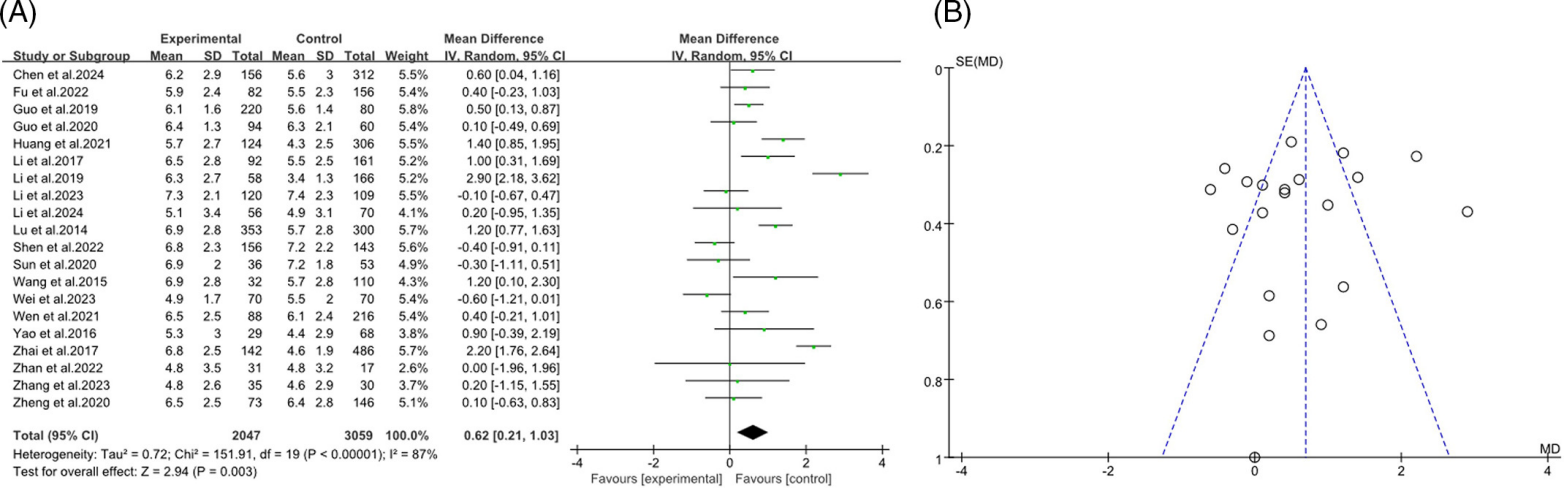
**(A)** Estimated MD summary for age. **(B)** Funnel plot for publication bias risk of age.

The forest plot presented a meta-analysis examining the impact of age on the incidence of RMPP. The analysis included 20 studies (9, 12–14, 16, 18, 19, 21–24, 26, 28–33, 35) with a total of 2,047 participants in the RMPP group and 3,059 in the NRMPP roup. The overall mean difference in age between the groups was 0.62 (*95% CI*: 0.21, 1.03), favoring the RMPP group. Significant heterogeneity was observed (*Tau*^2^ = 0.72; *Chi*^2^ = 151.91, *df* = 19, *P* *<* 0.00001; *I*^2^ = 87%), indicating variability in results across studies. The overall effect was significant (*Z* = 2.94, *P* = 0.003), suggesting that age was a relevant factor in RMPP incidence (Figure 2A). This funnel plot assessed publication bias for the relationship between age and RMPP in children. The symmetrical distribution of points around the vertical line suggested low risk of publication bias, indicating that the study results were likely reliable (Figure 2B).

#### 3.3.2 Gender

**Figure 3:**
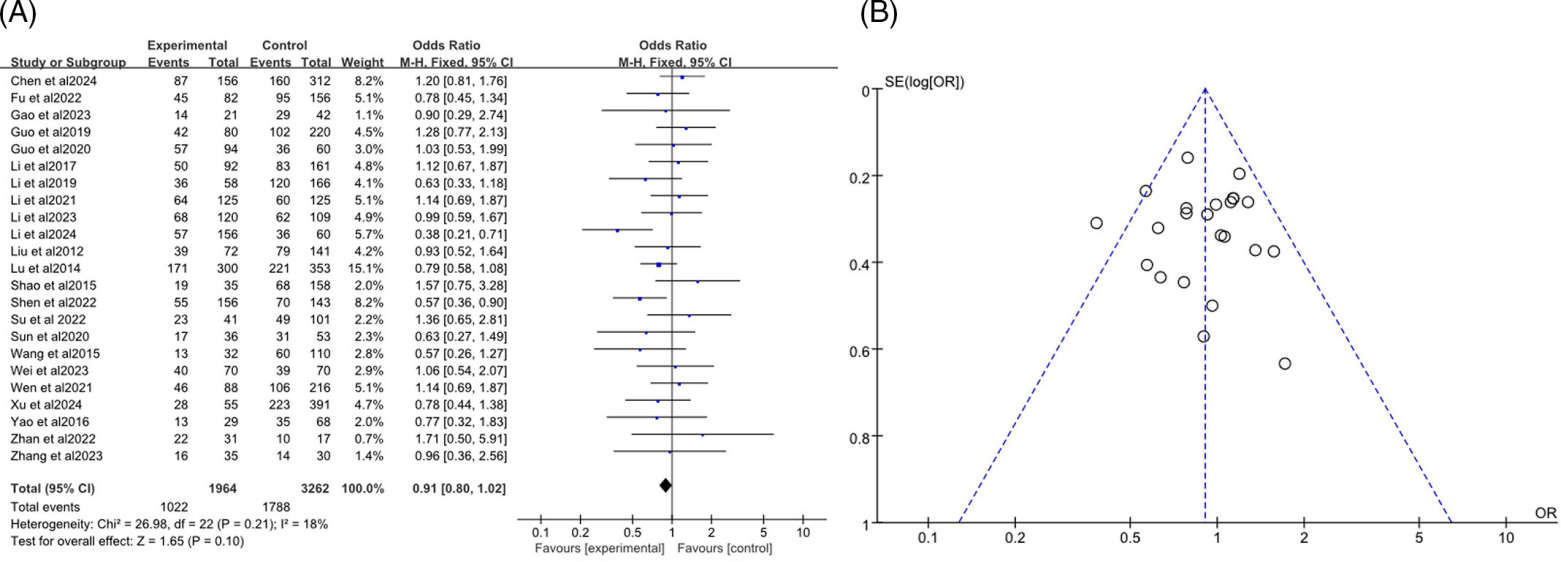
**(A)** Estimated OR summary for gender. **(B)** Funnel plot for publication bias risk of gender.

The analysis included a total of 23 studies (7–24, 27, 34–37), with 1,964 participants in the RMPP group and 3,262 in the NRMPP group. The overall odds ratio (*OR*) was 0.91 (*95% CI*: 0.80, 1.02), indicating no significant association between gender and the incidence of RMPP. The heterogeneity test (*Chi*^2^ = 26.98, *df* = 22, *P* = 0.21) indicated no significant heterogeneity among studies, and the overall effect test (*Z* = 1.65, *P* = 0.10) showed no significant overall effect (Figure 3A). The funnel plot in this meta-analysis displayed the distribution of studies based on their effect size (odds ratio) and sample size (indicated by the weight). The plot showed a relatively symmetrical distribution of studies around the overall effect size, suggesting low risk of publication bias (Figure 3B).

#### 3.3.3 CRP

**Figure 4:**
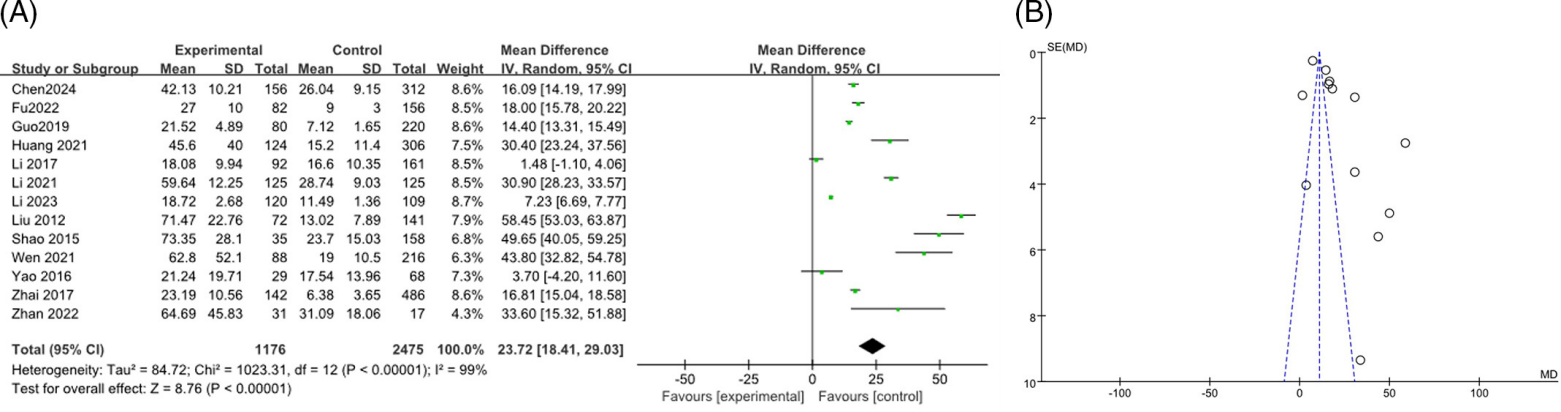
**(A)** Estimated MD summary for CRP. **(B)** Funnel plot for publication bias risk of CRP.

A total of 13 studies (9, 13, 15–17, 19, 21, 28, 34–37) reported the association between CRP levels and RMPP, including 3,651 cases. The heterogeneity results showed an *I*^2^ of 99%, *P* < 0.00001, indicating high heterogeneity among the studies. The random effects model was used for meta-analysis. The results indicated that the CRP levels in the RMPP group were higher than those in the NRMPP group, and the difference was statistically significant [*MD* (*95% CI*): 23.72 (18.41, 29.03), *P* < 0.001]. The funnel plot indicated a potential risk of publication bias. The points were mostly clustered around the center, but there were a few points that deviated significantly from the central line. This suggested that smaller studies with more significant effects might have been more likely to be published, while smaller studies with non—significant or negative results might have been less likely to be published. These findings are shown in Figures 4A,B. The subgroup analysis of the CRP outcome by age range (<12 years vs. ≥12 years) showed that in the ≥12 years subgroup, CRP in the RMPP group was significantly higher than that in the NRMPP group (with a stable effect), while in the <12 years subgroup, the results should be interpreted with caution due to high heterogeneity. Both the overall and subgroup heterogeneities were extremely strong, and age had a significant modifying effect on the CRP effect under the Common effect model (Supplementary Figure S1).

#### 3.3.4 LDH

**Figure 5:**
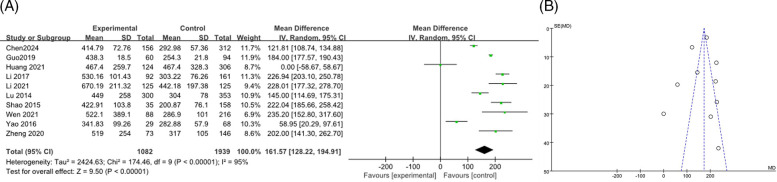
**(A)** Estimated MD summary for LDH. **(B)** Funnel plot for publication bias risk of LDH.

A total of 10 articles (9, 12, 14–16, 18, 19, 26, 28, 36) were combined to analyze LDH, including 3,021 patients, the heterogeneity test after merging showed statistical significance (*I*^2^ = 95%). The results of the random effects model showed that the LDH levels in the RMPP group were higher than those in the NRMPP group, and the difference was statistically significant [*MD* (*95% CI*): 161.57 (128.22, 194.91), *P* < 0.001], as shown in Figure 5A. The funnel plot showed asymmetry, indicating a potential risk of publication bias. The points were unevenly distributed around the central line, with more studies having positive results. This suggested possible underrepresentation of studies with negative or non-significant findings (Figure 5B). The subgroup analysis of the LDH outcome stratified by age range revealed that, in both subgroups, the LDH levels in the RMPP group were significantly elevated compared to those in the NRMPP group (with extremely high heterogeneity), and age exerted a significant modifying effect on the LDH response under the Common effect model (with more marked differences observed in the ≥12 years subgroup). (Supplementary Figure S2).

#### 3.3.5 Combination of CRP and LDH levels

**Figure 6:**
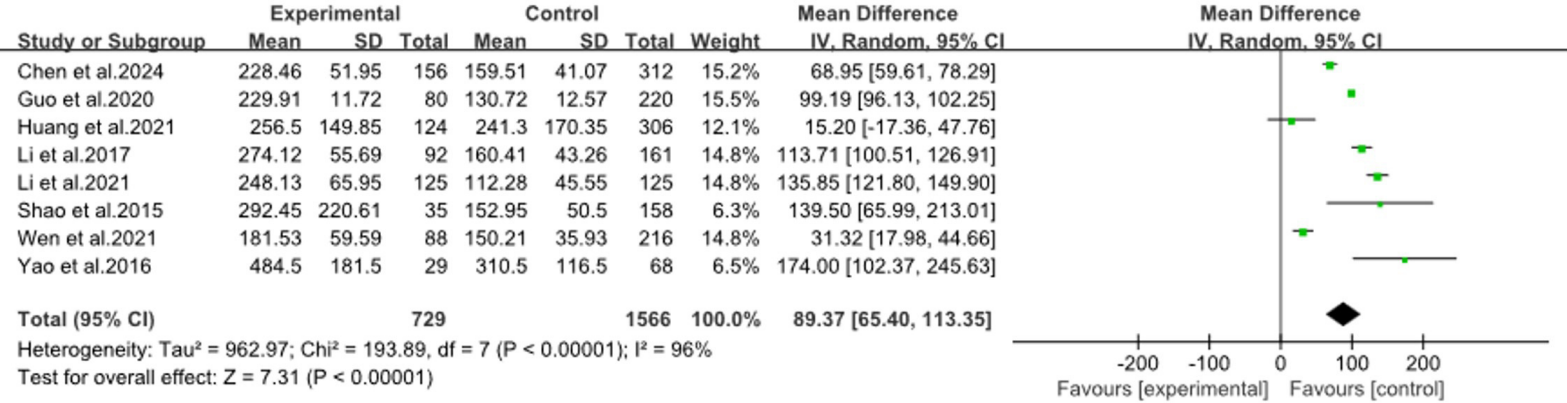
Estimated MD summary for combination of CRP and LDH levels.

The forest plot (Figure 6) showed a meta-analysis using combined CRP and LDH levels to predict RMPP in children. It included eight studies (9, 14–16, 19, 26, 28, 36), and found a significant mean difference of 89.37 (*95%CI*: 65.40, 113.35) favoring the RMPP group, with high heterogeneity (*I*^2^ = 96%). This indicated that while the combined markers were effective, the results varied widely across studies, suggesting a need for further research to refine the risk model.

#### 3.3.6 WBC

**Figure 7:**
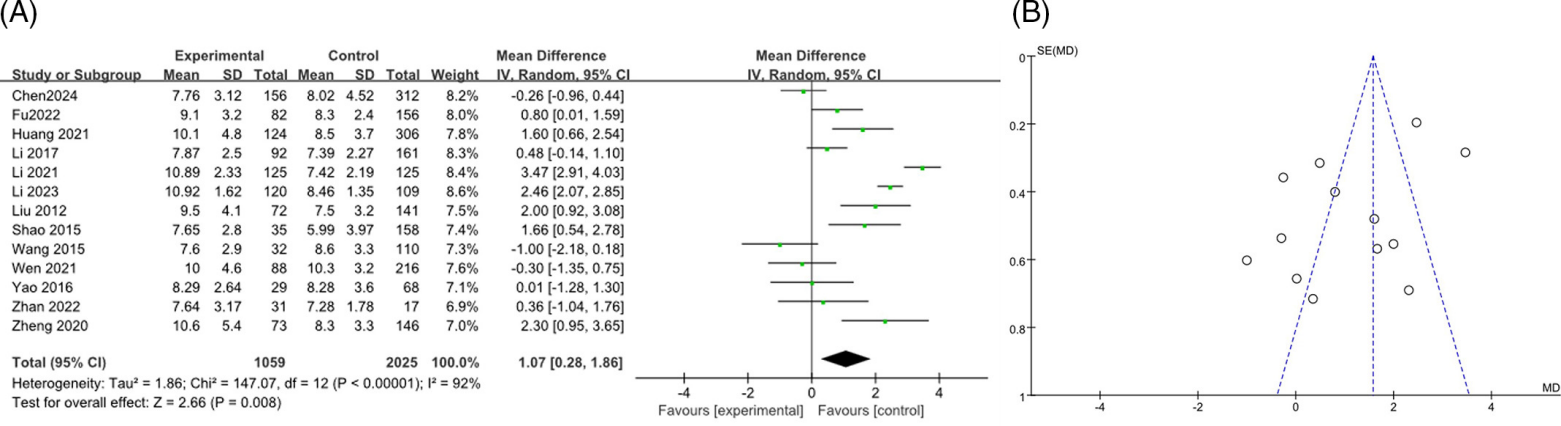
**(A)** Estimated MD summary for WBC. **(B)** Funnel plot for publication bias risk of WBC.

A total of 13 studies on WBC (9, 12, 15–17, 19, 21, 23, 26, 28, 34, 36) were merged and analyzed, including 3084 patients. The heterogeneity test after merging showed statistical differences (*I*^2^ = 92%). Therefore, a random effects model was used. The results showed that the WBC levels in the RMPP group were slightly higher than those in the NRMPP group, and the difference was statistically significant [*MD* (*95% CI*): 1.07 (0.28, 1.86), *P* = 0.008] (Figure 7A). The funnel plot appeared symmetrical, suggesting a low risk of publication bias (Figure 7B). The subgroup analysis of the WBC outcome stratified by age range demonstrated that, in the ≥12 years subgroup, the WBC levels in the RMPP group remained significantly higher than those in the NRMPP group under the random effects model, whereas in the <12 years subgroup, the results from the random effects model were unstable due to high heterogeneity. Additionally, age exerted a significant modifying effect on the WBC response under the fixed effects model, with more prominent differences observed in the ≥12 years subgroup. (Supplementary Figure S3).

#### 3.3.7 Neutrophils (%)

**Figure 8:**
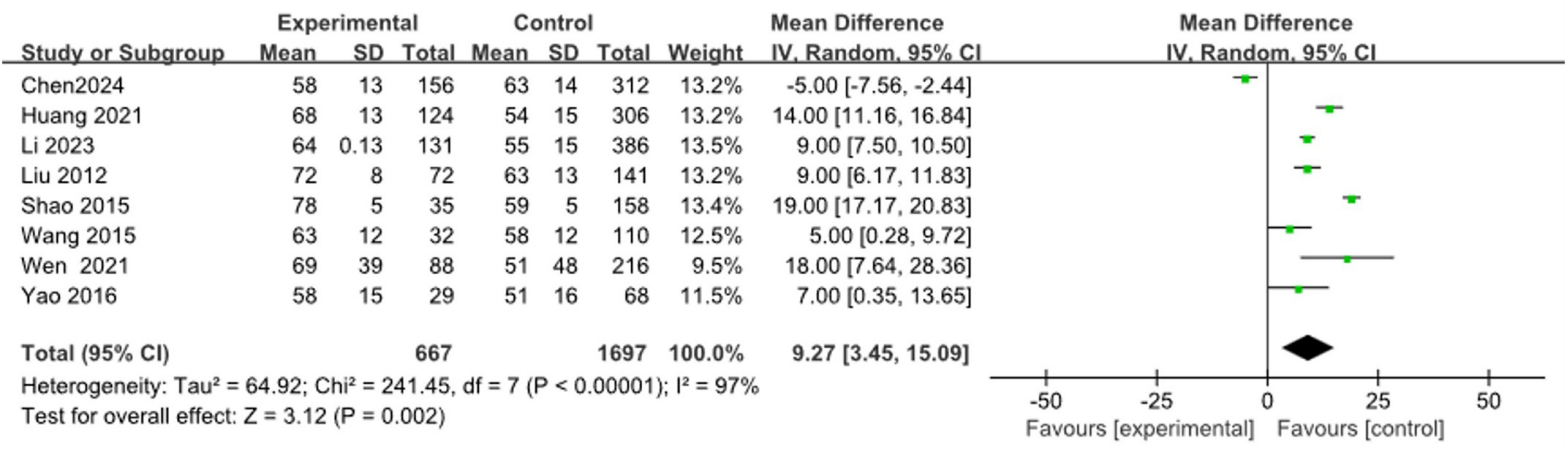
Estimated MD summary for neutrophils (%).

Eight articles (9, 19, 23, 26, 28, 31, 34, 36) were combined to analyze Neutrophils (%), including 2,364 cases. The heterogeneity test after merging was statistically signiﬁcant (*I*^2^ = 97%). The results found that the Neutrophils (%) of the RMPP group were significantly higher than those of the NRMPP group, and the difference was statistically significant [*MD* (*95%CI*): 9.27 (3.45, 15.09), *P* = 0.002] (Figure 8).

#### 3.3.8 Fever duration (days)

**Figure 9:**
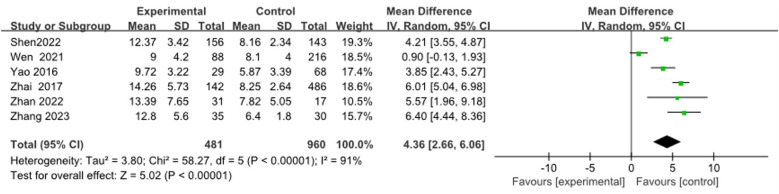
Estimated MD summary for fever duration (days).

Six articles (9, 13, 19, 26, 29, 30) were merged to analyze the duration of fever, including 1,441 cases. Heterogeneity tests after merging showed poor homogeneity in the included literature (*I*^2^ = 91%). Finally, the random effects model was adopted. The study suggested that the duration of fever in the RMPP group was longer than that in the NRMPP group, and the difference was statistically significant [*MD* (*95% CI*): 4.36 (2.66, 6.06), *P* < 0.001], as shown in Figure 9.

#### 3.3.9 AST

**Figure 10:**
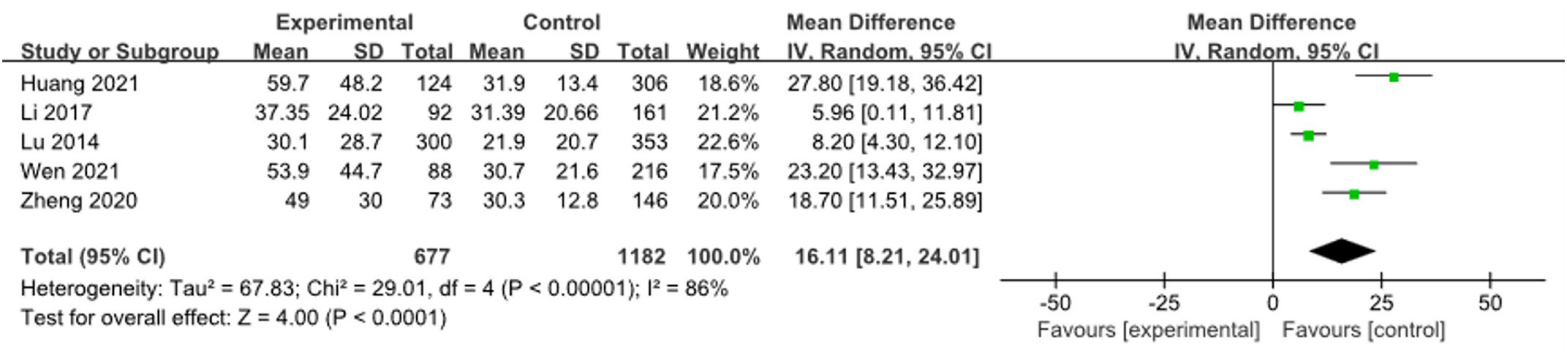
Estimated MD summary for AST.

After using a random effects model to summarize the AST levels of 5 studies (9, 12, 16, 18, 19), it was found that the AST levels in the RMPP group were significantly higher than those in the NRMPP group [*MD* (*95% CI*): 16.11 (8.21, 24.01), *P* < 0.001], as shown in Figure 10.

#### 3.3.10 ALT

**Figure 11:**
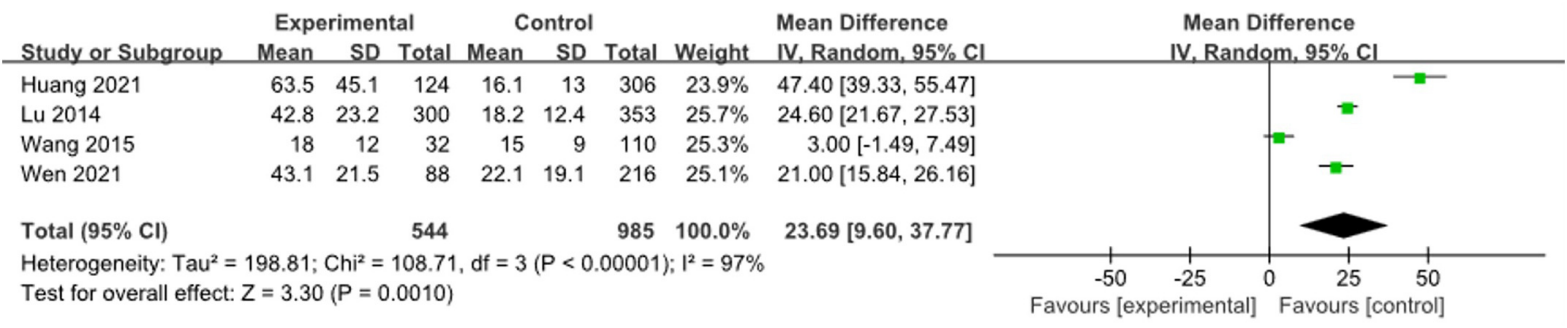
Estimated MD summary for ALT.

Four studies (9, 12, 16, 23) were combined and analyzed for ALT, including 1,529 cases. The analysis indicated that the ALT levels in the RMPP group were significantly higher than those in the NRMPP group [*MD* (*95% CI*): 23.69 (9.60, 37.77), *P* = 0.001] (Figure 11).

#### 3.3.11 IL-6

**Figure 12:**
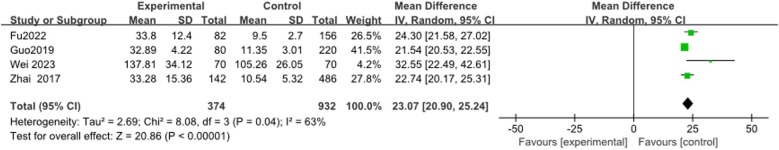
Estimated MD summary for IL-6.

Four studies (13, 21, 24, 35) reported IL-6 levels, with heterogeneity results showing *I*^2^ = 63%. Meta-analysis showed that IL-6 levels in the RMPP group were significantly higher than those in the NRMPP group [*MD* (*95% CI*): 23.07 (20.90, 25.24), *P* < 0.0001] (Figure 12).

#### 3.3.12 ESR

**Figure 13:**
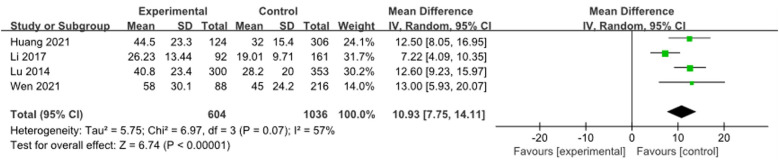
Estimated MD summary for ESR.

A total of 4 studies (9, 16, 18, 19) were combined to analyze ESR, including 1,640 patients. Heterogeneity tests showed an *I*^2^ of 57%, and meta-analysis results showed that the ESR of the RMPP group was higher than that of the NRMPP group [*MD* (*95% CI*): 10.93 (7.75, 14.11), *P* < 0.001], as shown in Figure 13.

#### 3.3.13 Combined pleural effusion

**Figure 14:**
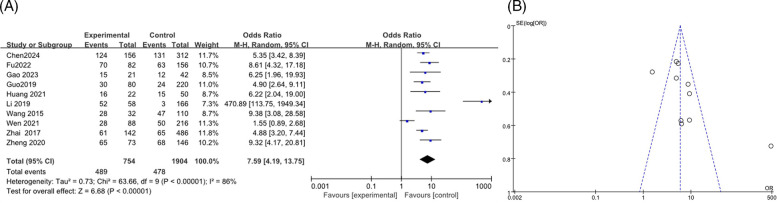
**(A)** Estimated OR summary for combined pleural effusion. **(B)** Funnel plot for publication bias risk of combined pleural effusion.

A total of 10 articles (12, 13, 19–21, 23, 27, 28, 33, 35) were analyzed, including 2,658 cases, with heterogeneity testing indicated an *I*^2^ of 86%. Therefore, the results of the random effects model indicated that the combined pleural effusion in the RMPP group was higher than that in the NRMPP group, and the difference was statistically significant [*OR* (*95% CI*): 7.59 (4.19, 13.75), *P* < 0.0001] as shown in the figure (Figure 14A). The funnel plot indicated potential publication bias, with data points being asymmetrically distributed around the center line, suggesting that studies with higher odds ratios were more likely to be published (Figure 14B).

#### 3.3.14 Lung consolidation

**Figure 15:**
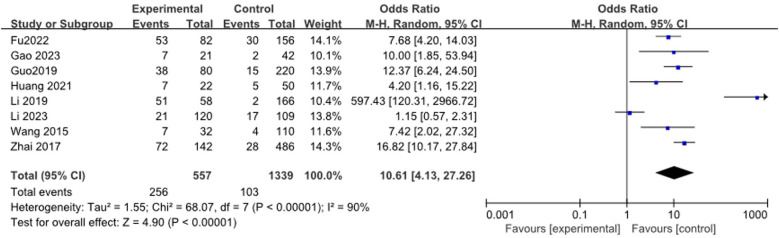
Estimated OR summary for lung consolidation.

A total of 8 studies (13, 17, 20, 21, 23, 27, 33, 35) reported the relationship between lung consolidation and RMPP, including 1,896 patients. The heterogeneity results were statistically significant (*I*^2^ = 90%). Meta-analysis showed that lung consolidation was a risk factor for RMPP in children[*OR* (*95% CI*): 10.61 (4.13, 27.26), *P* < 0.0001], as shown in Figure 15.

### 3.4 Sensitivity analysis

Sensitivity analysis was performed on factors with high heterogeneity through leave-one-out method. Results showed that heterogeneity for ESR, Fever duration, and Lung Consolidation was reduced after respectively removing studies (9, 19, 33). However, heterogeneity for LDH, WBC, IL-6, AST, ALT, Neutrophils (%), CRP, and Combined pleural effusion remained largely unchanged. These findings indicated the stability and high credibility of the results. The detailed results of the sensitivity analysis are presented in Supplementary Table S3.

### 3.5 Publication bias

Egger test was used to evaluate publication bias. The results indicated that WBC AST, ALT, CRP, IL-6, ESR, the duration of fever, and pleural effusion showed no significant publication bias, while LDH, neutrophil percentage, and lung consolidation showed evidence of publication bias (Supplementary Table S4). The Trim and Fill analysis indicated that for studies related to LDH, Neutrophil Percentage, and Pulmonary Consolidation, there was a low risk of publication bias. The effect sizes remained unchanged after adjustment, and the original results demonstrated high robustness, being not significantly affected by publication bias (Supplementary Figures S4–S6).

## 4 Discussion

To our knowledge, mycoplasma infection was an important component of community-acquired pneumonia in children, with an infection rate of 40% and a mortality rate of 1.38% (7, 27) during the epidemic. With the widespread use of macrolide antibiotics in recent years, the antibiotic resistance rate of *M. pneumoniae* had been rising, and the incidence rate of RMPP had also been increasing, especially in Asian countries (38–40). Children with RMPP could develop complications such as pleural effusion, bronchiectasis, and bronchiolitis obliterans, and their prognosis was often poor. Therefore, early identification and treatment of RMPP were extremely important for pediatricians (41). This study included a total of 28 articles, including 6,374 cases, which included a wider range of indicators compared to previous studies. These indicators included inflammatory factors and other features such as CRP, LDH, ESR, WBC, IL-6, neutrophil count (%), AST, ALT, duration of fever, lung consolidation, and pleural effusion.

The immune response to *M. pneumoniae*e infection played a pivotal role in the manifestation of clinical symptoms. CRP, ESR, LDH, and neutrophil count (%) were non-specific indicators of inflammatory response. CRP was an acute phase protein synthesized by liver cells when the body was subjected to inflammatory stimuli such as microbial invasion or tissue damage (15, 33). CRP increased several hours after the onset of inflammation and reached its peak within 48 h. The increase in CRP value lagged behind the change in inflammatory activity by about 12 h; however, it was important to detected earlier than clinical symptoms (23). LDH was an important enzyme that catalyzed the oxidation-reduction reaction between lactate and pyruvate in glycolysis and gluconeogenesis processes. It had highly sensitivity, and even mild tissue damage could cause changes in serum LDH levels. Therefore, LDH was an important factor reflecting the degree of tissue damage and disease (13, 14). After comprehensive analysis, we found that the levels of CRP and LDH in the RMPP group were significantly higher than those in the NRMPP group, which was consistent with previous studies (41). However, there were contradictions in current research regarding ESR, as studies showed that ESR was a risk factor for the development of RMPP (9, 23). While, some meta-analyses also found no difference in ESR between the two groups [MD (95% CI): 8.11 (−1.34,17.56), *P* = 0.09] (11). Our analysis once again confirmed that ESR was a risk factor for RMPP. Unlike CRP, ESR responded more slowly, typically becoming evident 2–3 days after the onset of the inflammatory response, and serves as a key marker for evaluating inflammatory reactions and disease activity (23). Study demonstrated that *M. pneumoniae*e infection of bronchial epithelial cells *in vitro* significantly altered cellular metabolism, characterized by increased glucose uptake, enhanced aerobic glycolysis, and augmented ATP synthesis. The synthesized ATP was released into the extracellular environment through vesicular exocytosis and pannexin channels, resulting in a marked increase in extracellular ATP levels. This elevated extracellular ATP interacted with cell surface receptors, activating inflammation—related signaling pathways such as P2X and P2Y receptors, leading to the release of inflammatory mediators and thus intensifying the inflammatory response (2). As the subgroup analysis suggested, older children, due to their relatively mature and overactive immune systems, were more prone to developing RMPP, a finding that was consistent with some reports (42, 43).

In clinical practice, we found that the WBC in children with *M. pneumoniae* infection was often within the normal range. Therefore, few pediatricians paid attention to the WBC in RMPP. Fortunately, through meta-analysis, we found that the WBC in the RMPP group was slightly higher than that in the NRMPP group [MD (95% CI): 1.15 (0.33, 1.97), *P* = 0.006]; This had rarely been mentioned in previous meta-analyses. The immune system generated a strong immune response during infection or inflammation, leading to an increase in WBC to counter potential threats (36). Our study also summarized the levels of AST, and ALT levels, which were rarely mentioned in previous meta-analyses. Some studies had also shown that multi-organ dysfunction was more severe in the RMPP group, especially liver dysfunction (18). Mycoplasmas possessed an array of virulence factors that enabled them to overcome numerous barriers and successfully invade the host's defenses. They achieved adhesion by binding to host cell receptors or the extracellular matrix via ligand proteins. During proliferation, mycoplasmas absorbed nutrients from host cells and released various metabolic byproducts, including hydrogen peroxide (H_2_O_2_), ammonia (NH_3_), and hydrogen sulfide (H_2_S), which caused local tissue damage (1). It should be noted that only four studies (9, 12, 16, 23) reported this, which limited its generalizability. After human were infected with pathogens, the occurrence and development of inflammation are mainly the result of the interaction between pro-inflammatory and anti-inflammatory cytokines. The body produced pro-inflammatory factors such as IL-6, TNF-α, and IFN-γ to drive away pathogens (13). Mycoplasmas may have secreted various exotoxins, such as hemolysins, and expressed multiple pathogenic enzymes. These enzymes, which included lipolytic enzymes, peptidases, phosphatases, ecto-ATPases, cytotoxic nucleases, and nucleotidases, contributed significantly to the toxicity affecting host cells. Additionally, some inherent components of mycoplasmas, like lipids, membrane lipoproteins, and even superantigens, could have had a considerable pathogenic impact on host cells or the immune system (1).

Studies indicated that RMPP had a longer fever duration, severe clinical symptoms, rapid progression of signs, often with large areas of lung involvement in a short period, prone to pleural effusion and atelectasis, prolonged course, poor response to macrolide antibiotics, and some cases were accompanied by systemic inflammatory response syndrome or severe extrapulmonary complications, and might even develop into severe pneumonia such as necrotizing pneumonia (44, 45). Study indicated that a fever lasting for more than 10 days was a risk factor for RMPP (46). In our study, the incidence of large-scale lung consolidation and pleural effusion in the RMPP group was significantly higher than that in NRMPP group, which was consistent with previous research. This may have been related to the increased permeability of alveolar and pleural capillaries caused by pulmonary infections and systemic inflammatory responses. The risk of pulmonary consolidation and pleural effusion in the RMPP group was significantly higher than that in the NRMPP group in our study. Some scholars proposed that if a patient experienced fever duration for over 7 days, CRP >110 mg/L, LDH > 478 U/L, and showed lung consolidation, it may have indicated the development of RMPP (45). This might have been related to the increased permeability of alveolar and pleural capillaries caused by pulmonary infections and systemic inflammatory responses. Small molecular proteins permeated the infected lung tissue through the alveolar capillary wall, and the exudate of proteinous edema fluid rapidly spreads through the alveoli, forming pulmonary consolidation. Extensive pulmonary consolidation involving the pleura led to a large amount of inflammatory pleural exudate (11).

In this study, except for the study by Xu D et al. (25), all other included studies were retrospective. They analyzed existing data, which might have been incomplete or inaccurately recorded, leading to the risk of incomplete predictors or the potential for ignoring predictors due to data deficiencies. Additionally, retrospective studies assessed outcomes after they had occurred, making the causal relationship between predictors and outcomes more uncertain. Furthermore, all studies included had used conventional logistic regression, which has limitations in model assumptions, high data demands, and weak handling of interactions and non-linear relationships. In contrast, prospective studies collect data and measure predictors before outcomes occur. This enhances model accuracy and reliability, and improves data integrity and consistency, effectively reducing bias risks. With the development of machine learning in the medical field, algorithms such as decision trees, support vector machines, and random forests have been applied to the construction of prediction models (47, 48). It is suggested that future research on pediatric RMPP risk prediction models should adopt prospective study methods, integrate machine learning techniques, and use large sample sizes to develop models with good risk performance and strong stability.

The limitations of this study were: (1) All participants in the study were from China, which might have impacted the extrapolation of the conclusions; (2) some exposure factors with high diagnostic value for RMPP (such as D-dimers, etc.) could not be processed and combined due to a lack of original studies; (3) The included studies were all case—control studies. This design limited the research depth and made various potential biases unavo- idable. (4) This meta- analysis used the Newcastle-Ottawa Scale (NOS) to assess study quality, which, while widely used, lacks the comprehensive bias assessment provided by tools like ROBINS-E. This may limit the detection of subtle biases, potentially affecting the robustness of our findings. Future research should consider using more detailed bias assessment tools to enhance the reliability of meta-analytic results.

## 5 Conclusions

In summary, the longer the fever duration in children, the more pronounced the increase in IL-6, CRP, LDH, ESR, and neutrophil (%), the more severe the liver function damage, and the more serious the pleural effusion, the more extensive the involvement of large areas of lung consolidation, and the more likely it was to complicate refractory *M. pneumoniae* pneumonia in children.

## Data Availability

The original contributions presented in the study are included in the article/Supplementary Material, further inquiries can be directed to the corresponding authors.
