# Supplementary material for: Risk factors for refractory Mycoplasma pneumoniae in Chinese children: a meta-analysis
Source: Front Pediatr. 2025 Jun 27;13:1512689. doi: 10.3389/fped.2025.1512689 (PMC12245905; doi:10.3389/fped.2025.1512689)
Supplement: Supplementary file 1 [file Supplementaryfile1.docx]

Supplementary Table 1:Search strategies of PubMed

| The steps | Search formula. |
| --- | --- |
| #1 | （child［MeSH Terms］）OR（children［Title/Abstract］） |
| #2 | （（refractory mycoplasma pneumoniae pneumonia［MeSH  Terms］）OR（rmpp［Title/Abstract］））OR（RMPP［Title/ Abstract］） |
| #3 | risk factors［MeSH Terms］ OR Risk Factors［Title/Abstract］ OR Factor，Risk［Title/Abstract］ OR Risk Factor［Title/ Abstract］ OR Social Risk Factors［Title/Abstract］ OR Factor，Social Risk［Title/Abstract］ OR Factors，Social Risk ［Title/Abstract］ OR Risk Factor，Social［Title/Abstract］ OR Risk Factors，Social［Title/Abstract］ OR Social Risk Factor［Title/Abstract］ OR Health Correlates［Title/Abstract］ OR Correlates，Health［Title/Abstract］ OR Population at Risk ［Title/Abstract］ OR Populations at Risk［Title/Abstract］ OR Risk Scores［Title/Abstract］ OR Risk Score［Title/Abstract］ OR Score，Risk［Title/Abstract］ OR Risk Factor Scores［Title/ Abstract］ OR Risk Factor Score［Title/Abstract］ OR Score， Risk Factor［Title/Abstract］ |
| #4 | #1 AND #2 AND #3 |

Supplementary Table 2:Methodological quality assessment of included studies

| Study | Selection(score) | Comparability (score) | Exposure/Outcome  Assessment(score) | Total score |
| --- | --- | --- | --- | --- |
|  |  |  |  |  |
| Xu et al.(29) | 3 | 2 | 3 | 8 |
| Chen et al.(33) | 4 | 2 | 3 | 9 |
| Li et al.(38) | 3 | 1 | 3 | 7 |
| Li et al.(37) | 3 | 1 | 3 | 7 |
| Wei et al.(28) | 3 | 2 | 3 | 8 |
| Gao et al.(28) | 3 | 1 | 3 | 7 |
| Zhang et al.(35) | 3 | 2 | 3 | 8 |
| Li et al.(18) | 3 | 1 | 3 | 7 |
| Fu et al.(23) | 3 | 2 | 3 | 8 |
| Su et al.(31) | 3 | 0 | 3 | 6 |
| Shen et al.(34) | 3 | 1 | 3 | 7 |
| Zhan et al.(39) | 3 | 2 | 3 | 8 |
| Li et al.(16) | 3 | 1 | 3 | 7 |
| Wen et al.(21) | 3 | 2 | 3 | 8 |
| Huang et al.(22) | 3 | 1 | 3 | 7 |
| Huang et al.(36) | 3 | 1 | 3 | 7 |
| Zheng et al.(12) | 3 | 1 | 3 | 7 |
| Guo et al.(14) | 3 | 1 | 3 | 7 |
| Sun et al.(26) | 3 | 2 | 3 | 8 |
| Li et al.(15) | 3 | 0 | 3 | 6 |
| Guo et al.(24) | 3 | 0 | 3 | 6 |
| Zhai et al.(13) | 3 | 2 | 3 | 8 |
| Li et al.(17) | 3 | 1 | 3 | 7 |
| Yao et al.(30) | 3 | 1 | 3 | 7 |
| Shao et al.(25) | 3 | 0 | 3 | 6 |
| Wang et al.(27) | 3 | 1 | 3 | 7 |
| Lu et al.(20) | 3 | 2 | 3 | 8 |
| Liu et al.(19) | 3 | 0 | 3 | 6 |

Supplementary Table3: Sensitivity analysis of risk factors for RMPP

| **Factors** | ***I***2 Value | P Value | ***SMD(OR) 95%CI*** |
| --- | --- | --- | --- |
| LDH | 97.3% | 0.000 | 1.346(0.811~1.880) |
| WBC | 90.5% | 0.010 | 0.414(0.101~0.727) |
| IL-6 | 97.3% | 0.000 | 2.293(1.165~3.422) |
| AST | 84.3% | 0.000 | 0.737 (0.416~1.058) |
| ALT | 88.0% | 0.000 | 1.399 (1.029~1.768) |
| Neutrophils (%) | 93.8% | 0.004 | 0.462 (0.070~0.855) |
| CRP | 97.7% | 0.000 | -2.423 (-3.056 ~-1.789) |
| ESR | 0.0% | 0.683 | -0.576 (-0.694~0.457) |
| Fever duration | 61.7% | 0.141 | -1.380 (-1.646~-1.114) |
| Lung Consolidation | 66.3% | 0.253 | 5.268 (3.560~7.796) |
| Combined pleural effusion | 79.7% | 0.050 | 14.528 (6.758 ~ 31.233) |

**
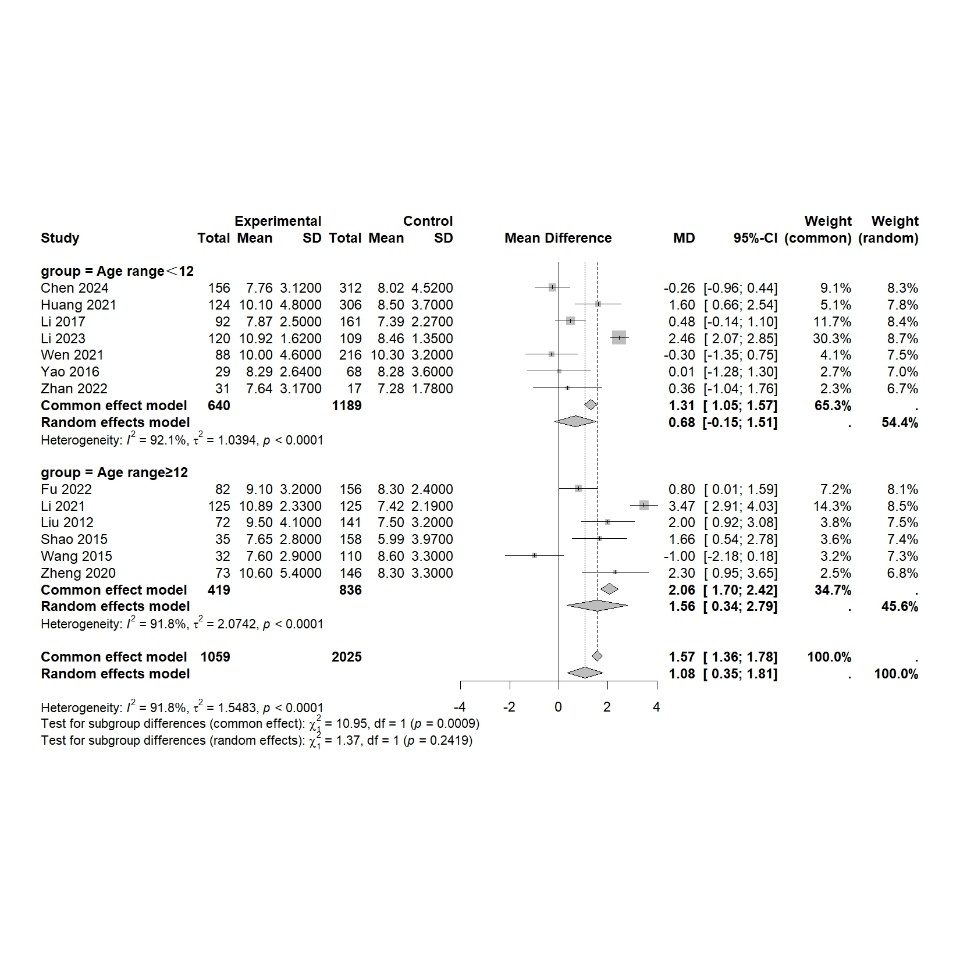
**

Supplementary Fig 1: The results of the stratified analysis of CRP by age range


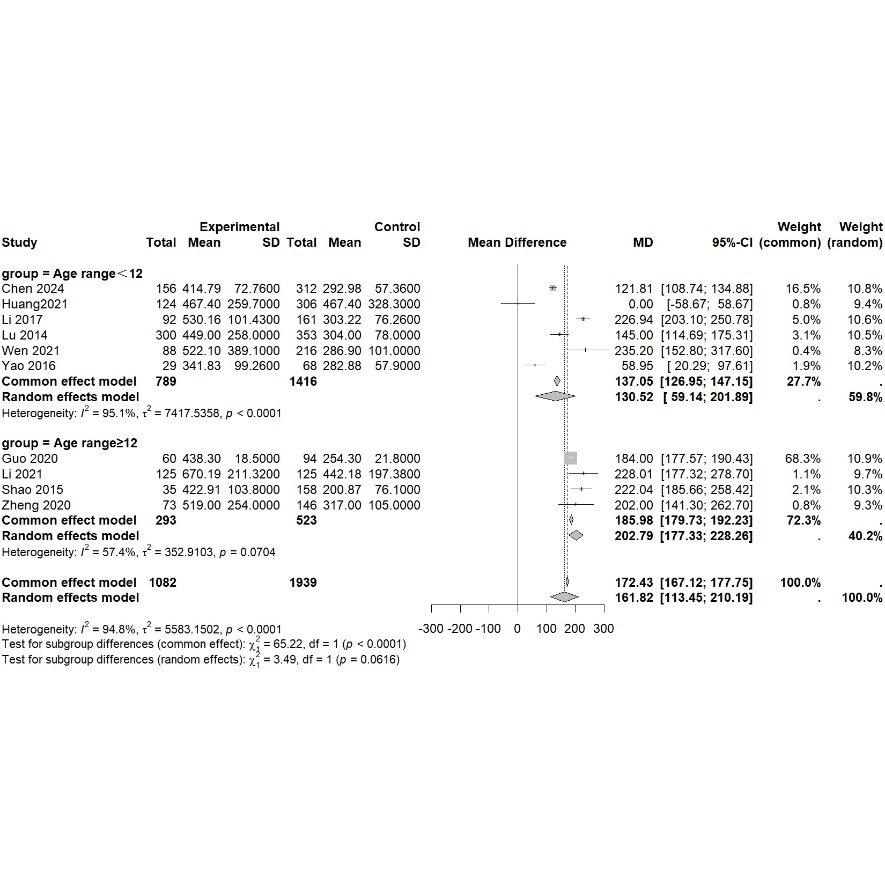


Supplementary Fig 2: The results of the stratified analysis of LDH by age range


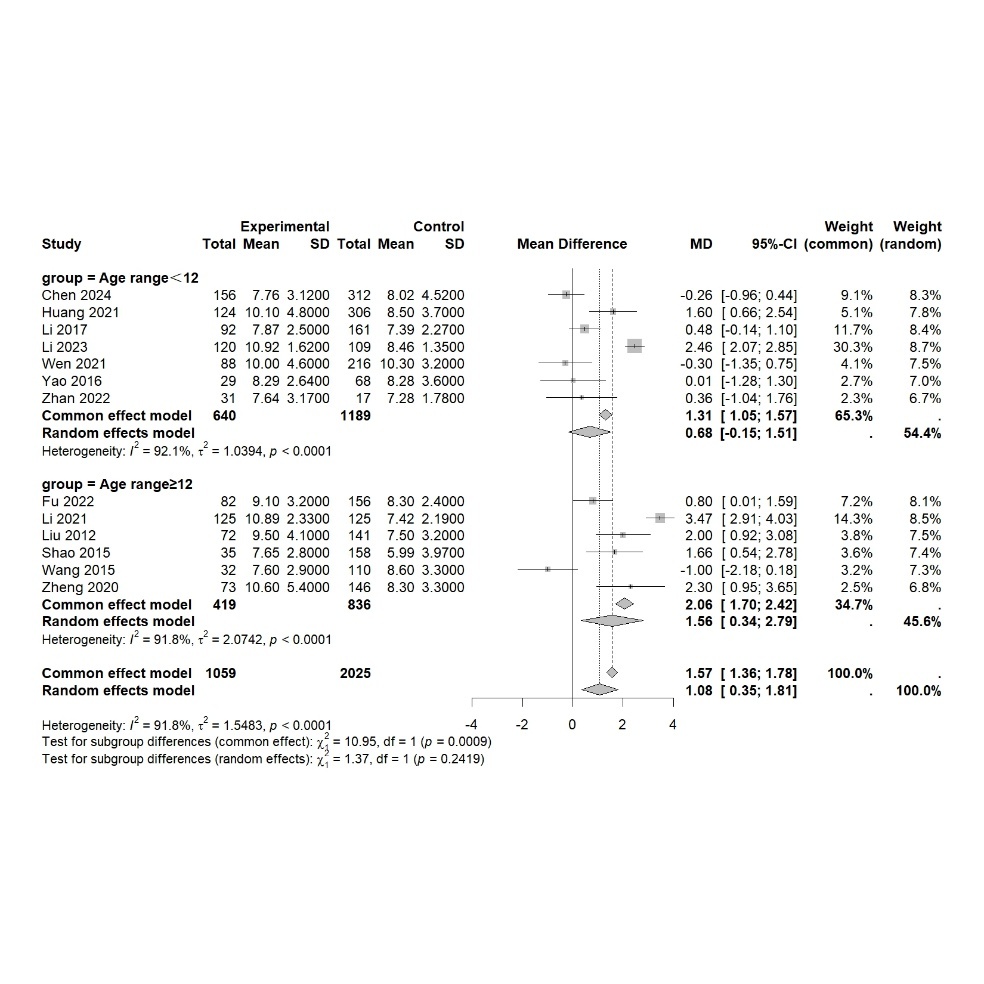


Supplementary Fig 3: The results of the stratified analysis of WBC by age range


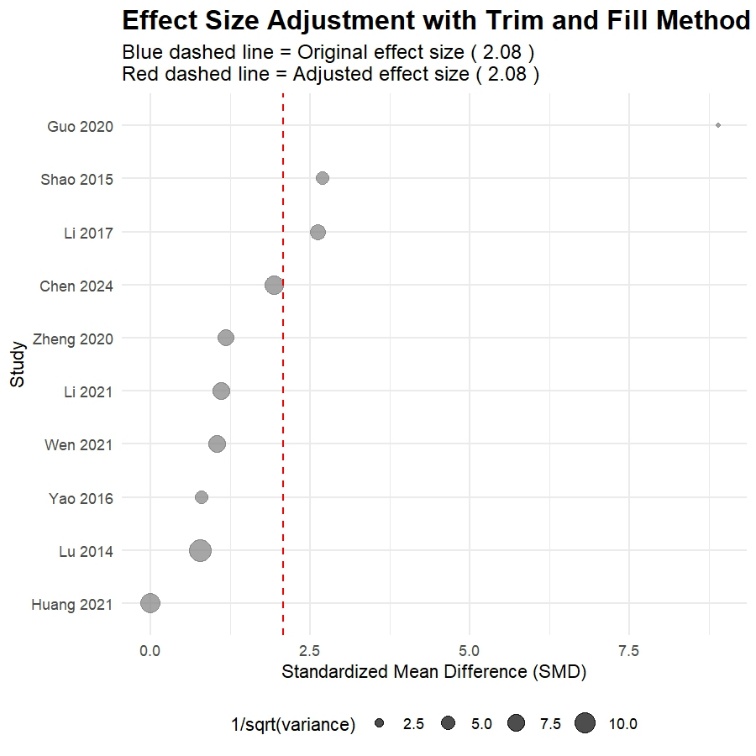


Supplementary Fig 4: Effect Size Adjustment with Trim and Fill Method for LDH


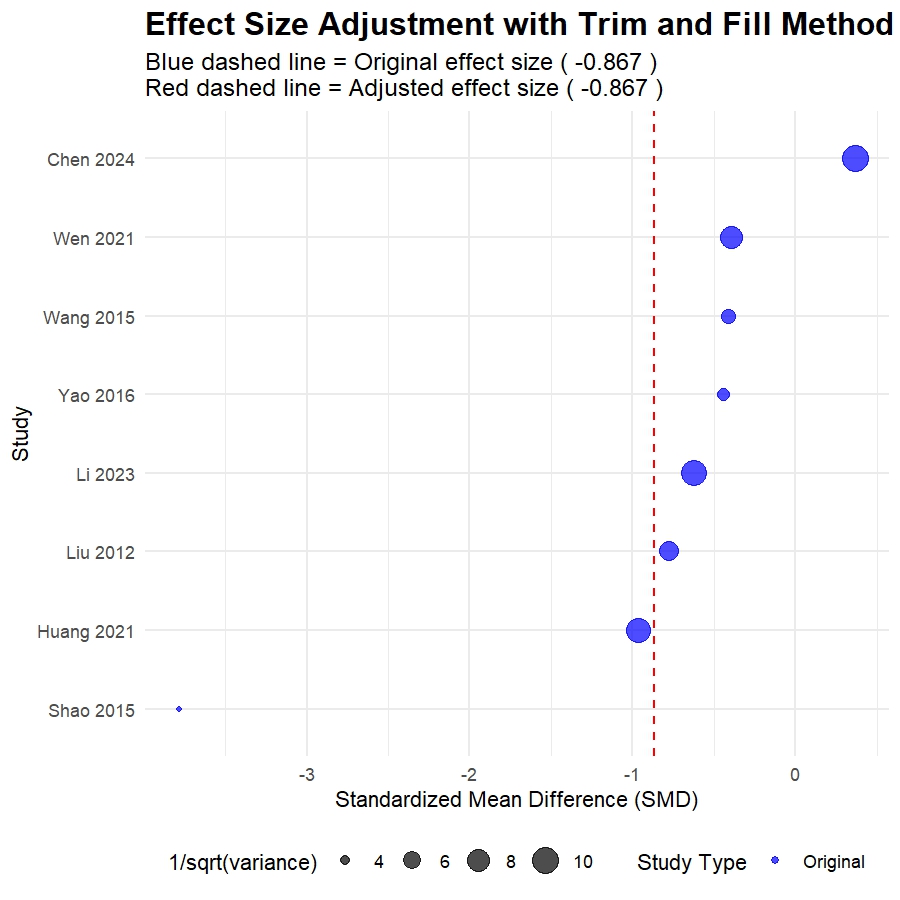


Supplementary Fig 5: Effect Size Adjustment with Trim and Fill Method for neutrophils (%)


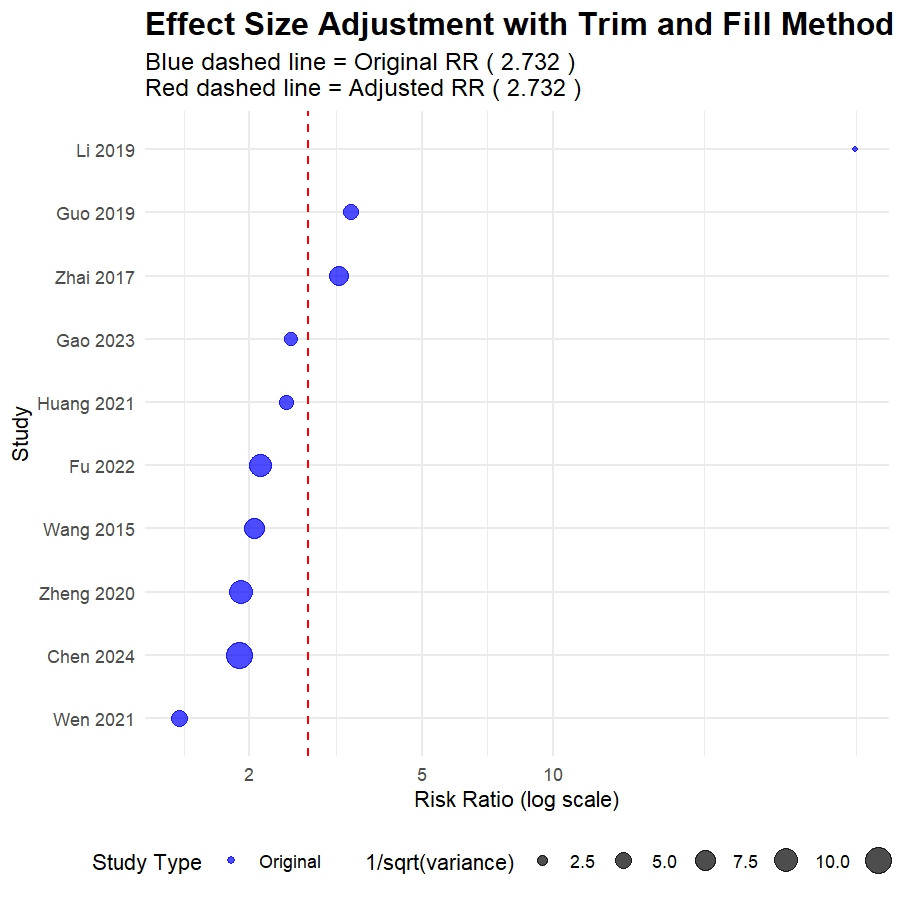


Supplementary Fig 6: Effect Size Adjustment with Trim and Fill Method for lung consolidation
